# Supplementary material for: Social learning of emotion and its implication for memory: an ERP study
Source: Sci Rep. 2026 Mar 14;16:9085. doi: 10.1038/s41598-026-42906-0 (PMC12993038; doi:10.1038/s41598-026-42906-0)

# Supplementary Information 1

**The timing for target image presentation to induce ‘perceptual uncertainty’**

The timing of the target image presentation – 27 ms – is chosen to create ‘perceptual uncertainty’ in judging the valence of the target images. Previously, it has been found that images presented for 20 ms are sufficient to detect the presence or absence of a particular target stimulus (animals) and that the visual processing of the target stimulus is reflected in the ERPs around 150 ms after stimulus onset ^111^. A recent study using drift-diffusion modeling suggests that a stimulus presentation duration of 25 ms with backward masking achieves conscious perception, with a mean accuracy close to 80% in detecting the valence categories of facial expressions - happy, sad, or neutral ^112^. Results from a visual awareness study indicated that at a stimulus presentation timing of 33 ms followed by backward masking, a considerable number of participants (64%) were able to detect the presence of an emotional target stimulus ^113^. However, the above cited studies used a stimulus presence/absence detection task or a valence classification task in facial expressions – which is relatively easier as compared to valence classification of images.

In the present study, we aim to creating perceptual uncertainty about the valence of the target images while consciously perceiving the target images on the screen. Hence, we chose a stimulus presentation duration of 27 ms but with a forward mask to allow the visual processing of the target image even in its absence. The chosen timing of stimulus presentation presented with the forward mask, will enable conscious perception of the target image while creating perceptual uncertainty in judging the valence of the target images. Additionally, the chosen timing also fits well with the frame rate of the monitor that we will use for stimulus presentation (144 Hz frame rate, 27 ms equals 4 frames) to achieve a better stimulus presentation precision.

In our design, we propose to use a forward mask rather than a backward mask followed by a blank screen to allow the visual processing of the briefly presented target image to take place. Since we are interested in the neural dynamics of the target image at both the early and later stages of processing, having a forward mask would introduce considerable ‘perceptual uncertainty’ in judging the valence of the target images, while still allowing the processing of the target image to take place during the blank screen, which can be captured by the ERP measures time-locked to the target image presentation.

Taken together, the cumulative effect of the timing of the brief stimulus presentation timing along with the forward mask will make social cues such as facial expressions highly relevant for valence judgments of the target images.

**Image selection criteria**

|  | **Mean Valence** | **Mean Arousal** | **Mean Complexity** |
| --- | --- | --- | --- |
| **Positive (N = 48)** | 6.96 (.03) | 4.72 (.06) | 2.10 (.07) |
| **Negative (N = 36)** | 2.55 (.08) | 5.67 (.08) | 2.29 (.06) |
| **Neutral (N = 41)** | 4.88 (.03) | 3.07 (.05) | 1.15 (.07) |

**Table S1.** Selected target stimuli with their mean Valence and Arousal ratings from the IAPS database (Lang et al., 2005), and mean complexity ratings from Bradley et al., (2007) study. Values in parentheses indicate standard errors.

**Figures**

**
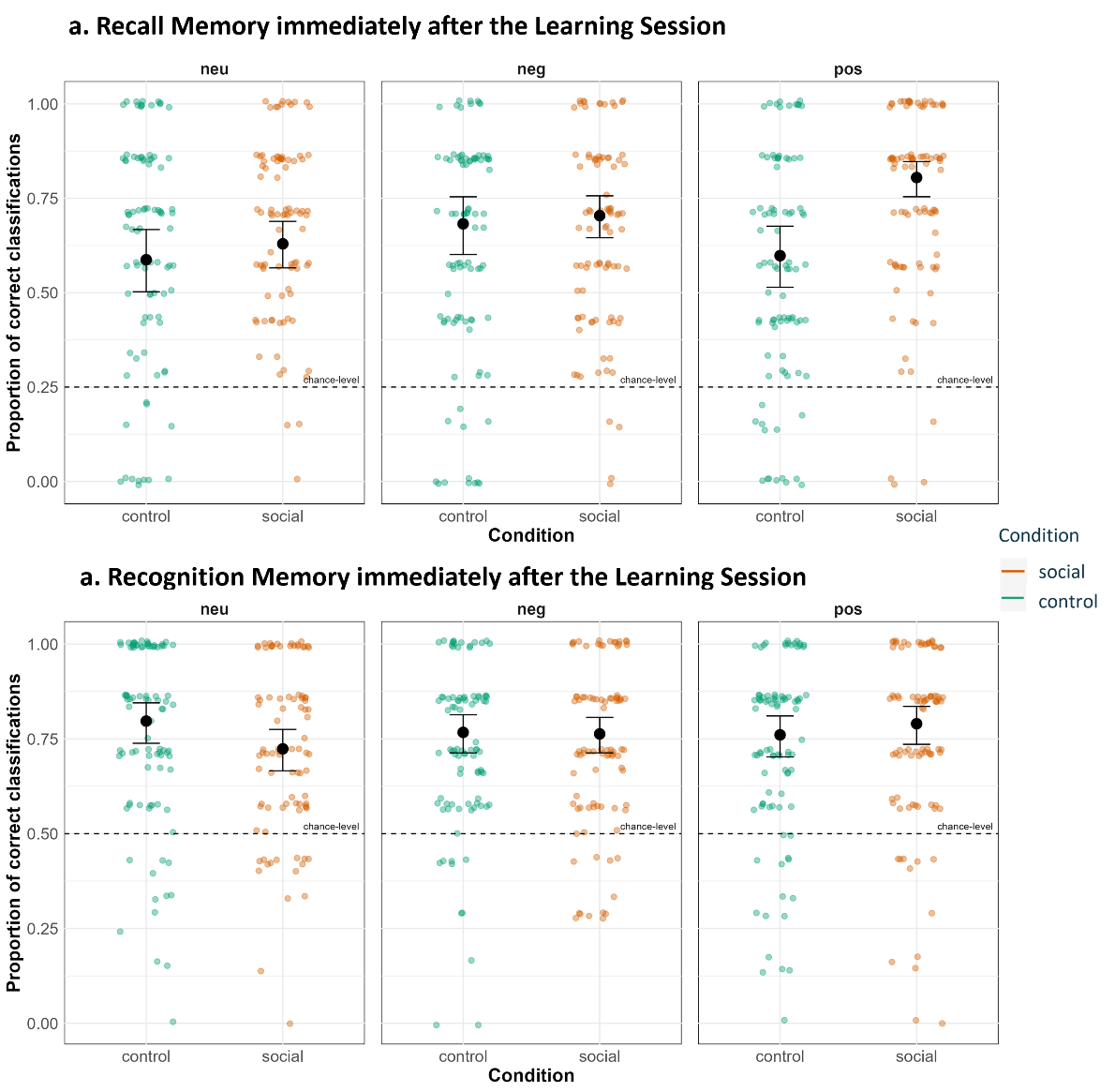
**

**Figure S1*.*** a. *Recall memory*. *b. Recognition memory.* Black dots indicate mean model-based predicted values along with the error bars indicating 95% CIs. Overlayed points represent mean accuracy values of individual participant per valence, per cue condition.


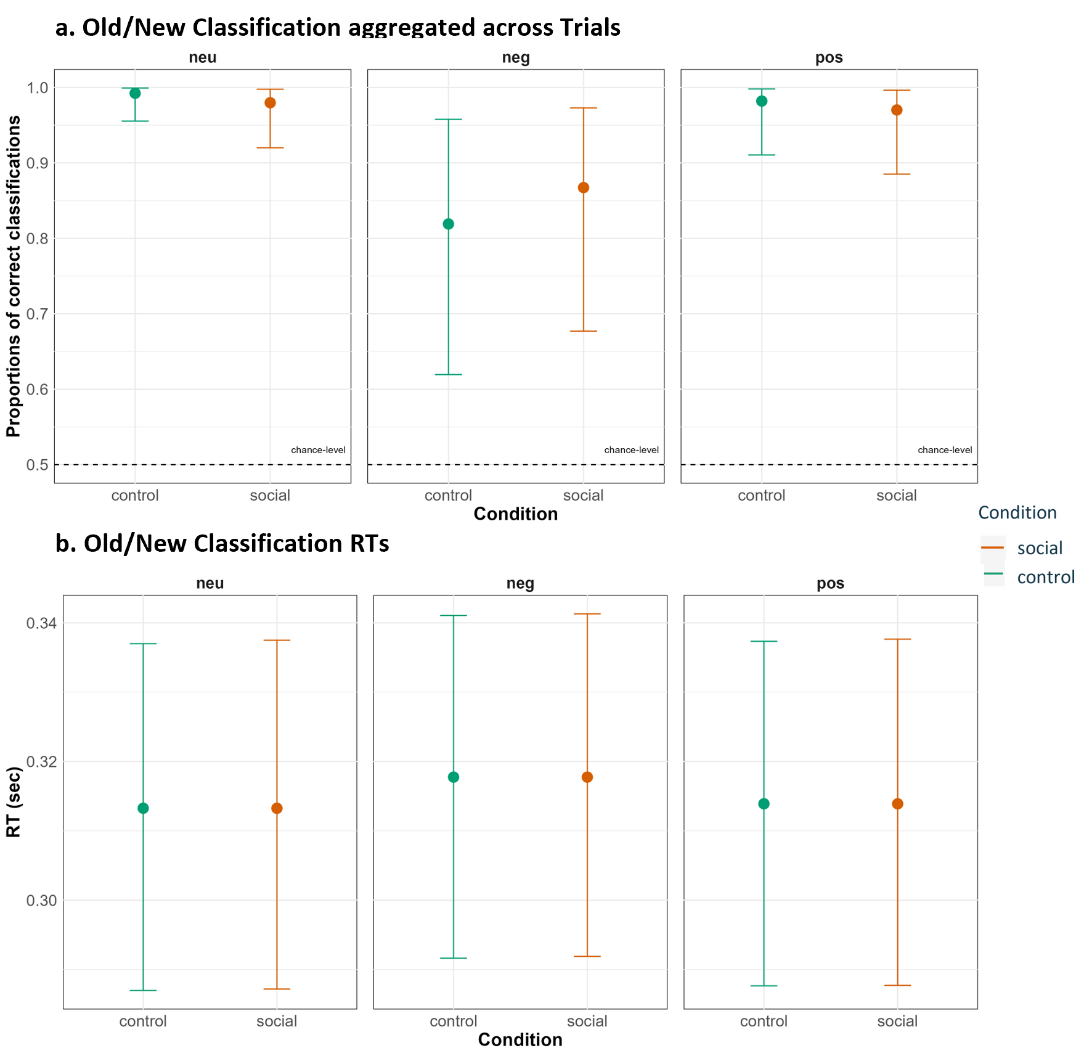


**Figure S2*.*** *a.* *Old/New Classification Accuracy aggregated across Trials*. *b. Old/New Classification RTs aggregated across Trials*. Plots represent mean model-based predicted values with error bars representing 95% CIs.


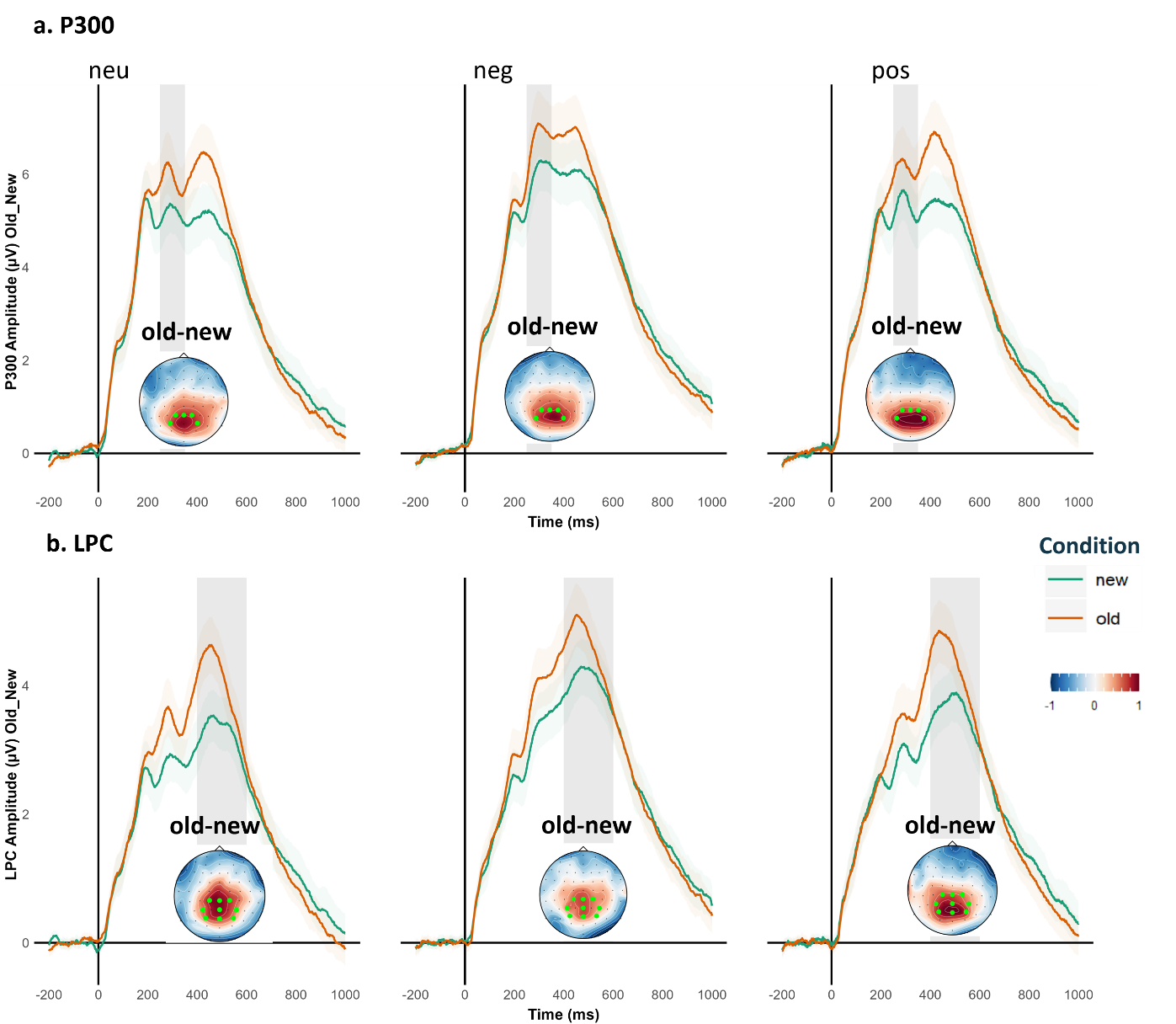


**Figure S3*.*** *a. P300. b. LPC*. Grand average waveforms across cue conditions for each of the target image valence category. Gray rectangular shaded region marks the time-window of the ERP. Topoplots indicate difference between cue conditions and green dots are the corresponding regions of interest (ROIs) for each of the ERPs.


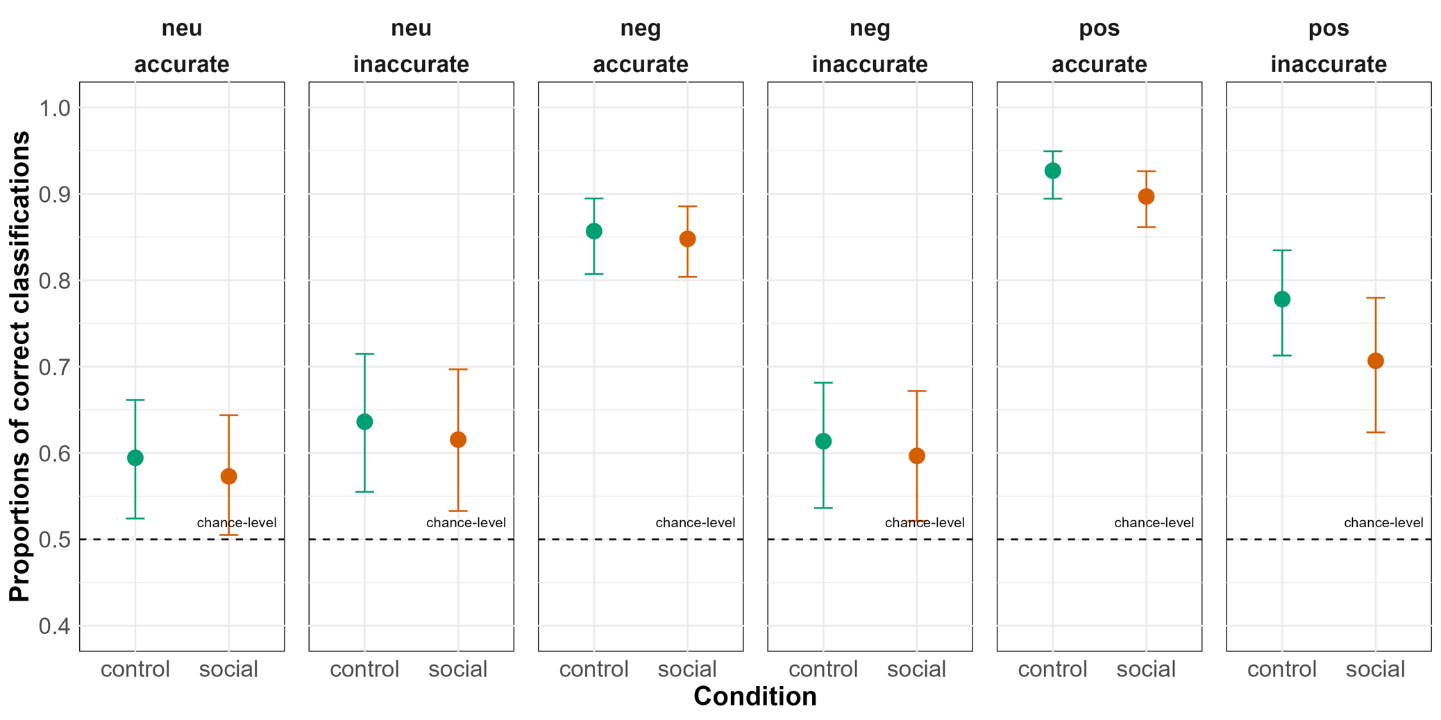


**Figure S4.**  *Old-New Classification Accuracy split by classification accuracy*. Plots represent mean model-based predicted values with their corresponding 95% CIs as error bars.

**Equivalence Testing**

To evaluate whether the non-significant cue condition (social/control) effect on the ERP amplitudes could be considered practically equivalent to zero, we conducted a two one-sided tests (TOST) procedure using the preregistered smallest effect size of interest (SESOI) of ±0.20 standardized units. This preregistered SESOI was defined in the Stage 1 protocol as the smallest theoretically meaningful effect size for early and mid-latency ERP amplitudes. In addition, we conducted an exploratory TOST analysis using a narrower SESOI of ±0.10 standardized units. This exploratory bound was selected because it reflects a more conservative criterion corresponding to minimal physiologically meaningful differences in ERP amplitudes, particularly given the relatively low amplitude range and high temporal precision of the components examined. This SESOI also reflects the smaller effects of ERP amplitudes previously found in literature.

**Day 1 Learning Session**

**EPN registered equivalence test (±0.20 standardized = ±1.20 µV):** To determine whether the non-significant cue condition effect for the EPN could be considered practically equivalent to zero, we conducted a preregistered TOST procedure using bounds of ±1.20 µV (corresponding to ±0.20 standardized units). The test indicated that the effect fell well within the equivalence region, t_lower (79) = 18.24, p < .001; t_upper (79) = –21.06, p < .001. Because both tests were significant at the Pocock-adjusted α = .0221, the cue condition effect can be interpreted as statistically equivalent to zero.

**EPN exploratory equivalence test (±0.10 standardized = ±0.60 µV):** We also conducted an exploratory TOST procedure using a narrower SESOI of ±0.60 µV (±0.10 standardized units). This test likewise supported equivalence, t_lower (79) = 8.41, p < .001; t_upper (79) = –11.23, p < .001, indicating that even under a conservative bound, the cue condition effect was negligible.

**LPC registered equivalence test (±0.20 standardized = ±1.05 µV):** To determine whether the non-significant cue condition effect for the LPC could be considered practically equivalent to zero, we conducted a preregistered TOST procedure using bounds of ±1.05 µV (corresponding to ±0.20 standardized units). The test indicated that the effect fell well within the equivalence region, t_lower (79) = 21.87, p < .001; t_upper (79) = –20.48, p < .001. Because both tests were significant at the Pocock-adjusted α = .0221, the cue condition effect can be interpreted as statistically equivalent to zero.

**LPC exploratory equivalence test (±0.10 standardized = ±0.53 µV):** We also conducted an exploratory TOST procedure using a narrower SESOI of ±0.53 µV (±0.10 standardized units). This test likewise supported equivalence, t_lower (79) = 11.28, p < .001; t_upper (79) = –9.89, p < .001, indicating that even under a conservative bound, the cue condition effect was negligible.

**Day 2 Test Session**

**P1 registered equivalence test (±0.20 standardized = ±1.51 µV):** To determine whether the non-significant cue condition effect for the P1 could be considered practically equivalent to zero, we conducted a preregistered TOST procedure using bounds of ±1.51 µV (corresponding to ±0.20 standardized units). The test indicated that the effect fell well within the equivalence region, t_lower (79) = 20.88, p < .001; t_upper (79) = –20.46, p < .001. Because both tests were significant at the Pocock-adjusted α = .0221, the cue condition effect can be interpreted as statistically equivalent to zero.

**P1 exploratory equivalence test (±0.10 standardized = ±0.75 µV):** We also conducted an exploratory TOST procedure using a narrower SESOI of ±0.75 µV (±0.10 standardized units). This test likewise supported equivalence, t_lower (79) = 10.54, p < .001; t_upper (79) = –10.12, p < .001, indicating that even under a conservative bound, the cue condition effect was negligible.

**LPC registered equivalence test (±0.20 standardized = ±1.01 µV):** To determine whether the non-significant cue condition effect for the LPC could be considered practically equivalent to zero, we conducted a preregistered TOST procedure using bounds of ±1.01 µV (corresponding to ±0.20 standardized units). The test indicated that the effect fell well within the equivalence region, t_lower (79) = 21.12, p < .001; t_upper (79) = –18.86, p < .001. Because both tests were significant at the Pocock-adjusted α = .0221, the cue condition effect can be interpreted as statistically equivalent to zero.

**LPC exploratory equivalence test (±0.10 standardized = ±0.50 µV):** We also conducted an exploratory TOST procedure using a narrower SESOI of ±0.50 µV (±0.10 standardized units). This test likewise supported equivalence, t_lower (79) = 11.12, p < .001; t_upper (79) = –8.87, p < .001, indicating that even under a conservative bound, the cue condition effect was negligible.

**Instructions given to the participants**

**Day1-Learning Session: Valence Classification Task**

**
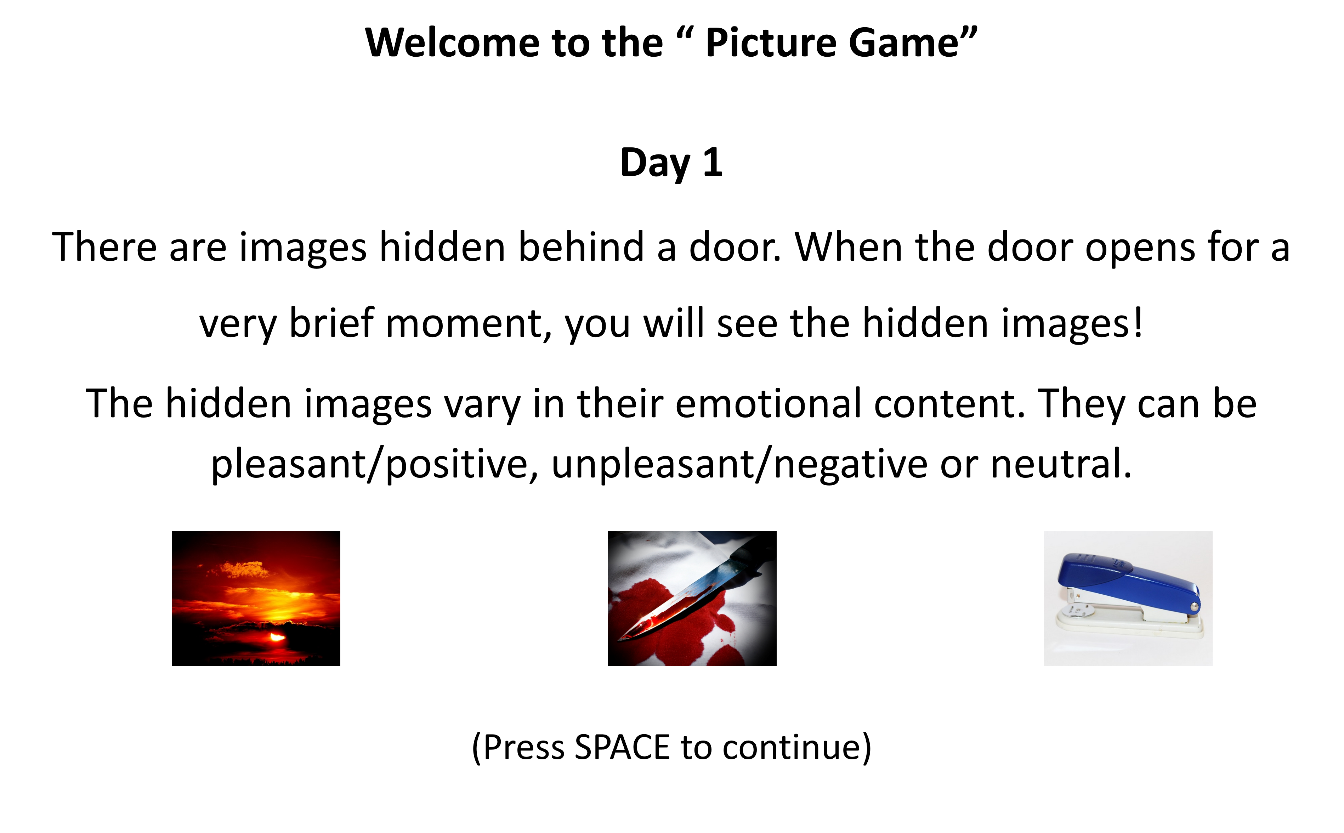
**

***The door mentioned in the instruction refers to the forward mask used in the experimental set-up**

**
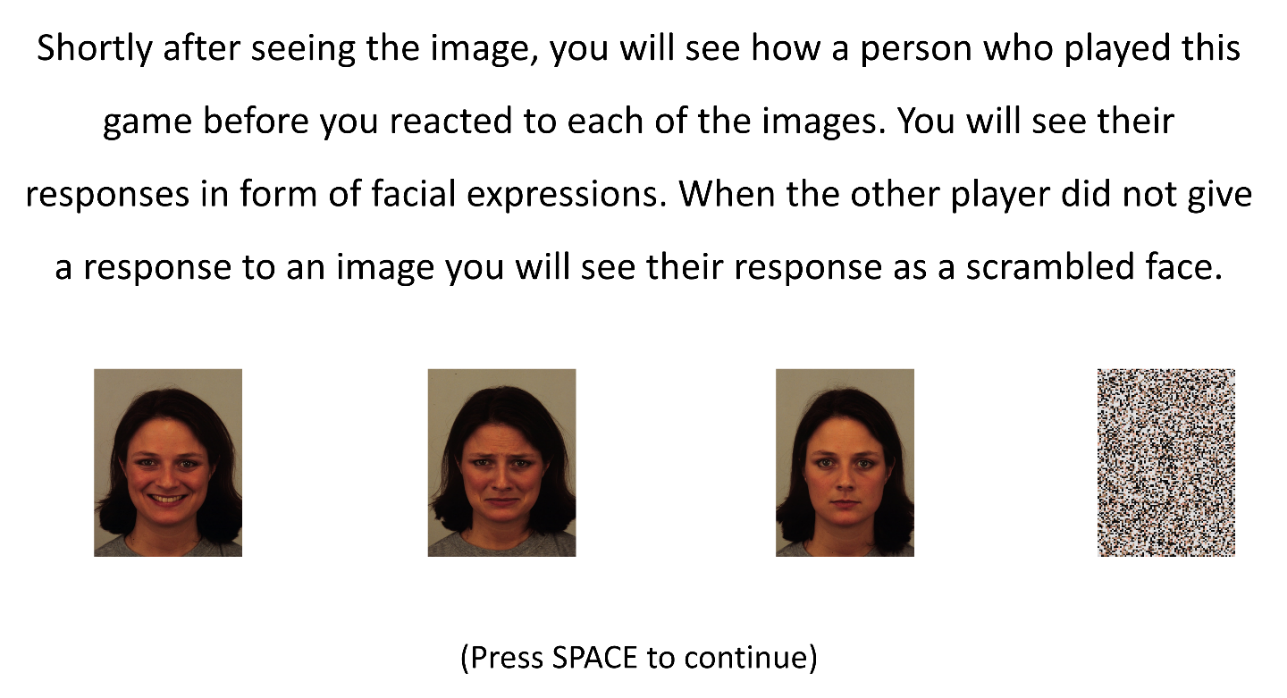
**

**
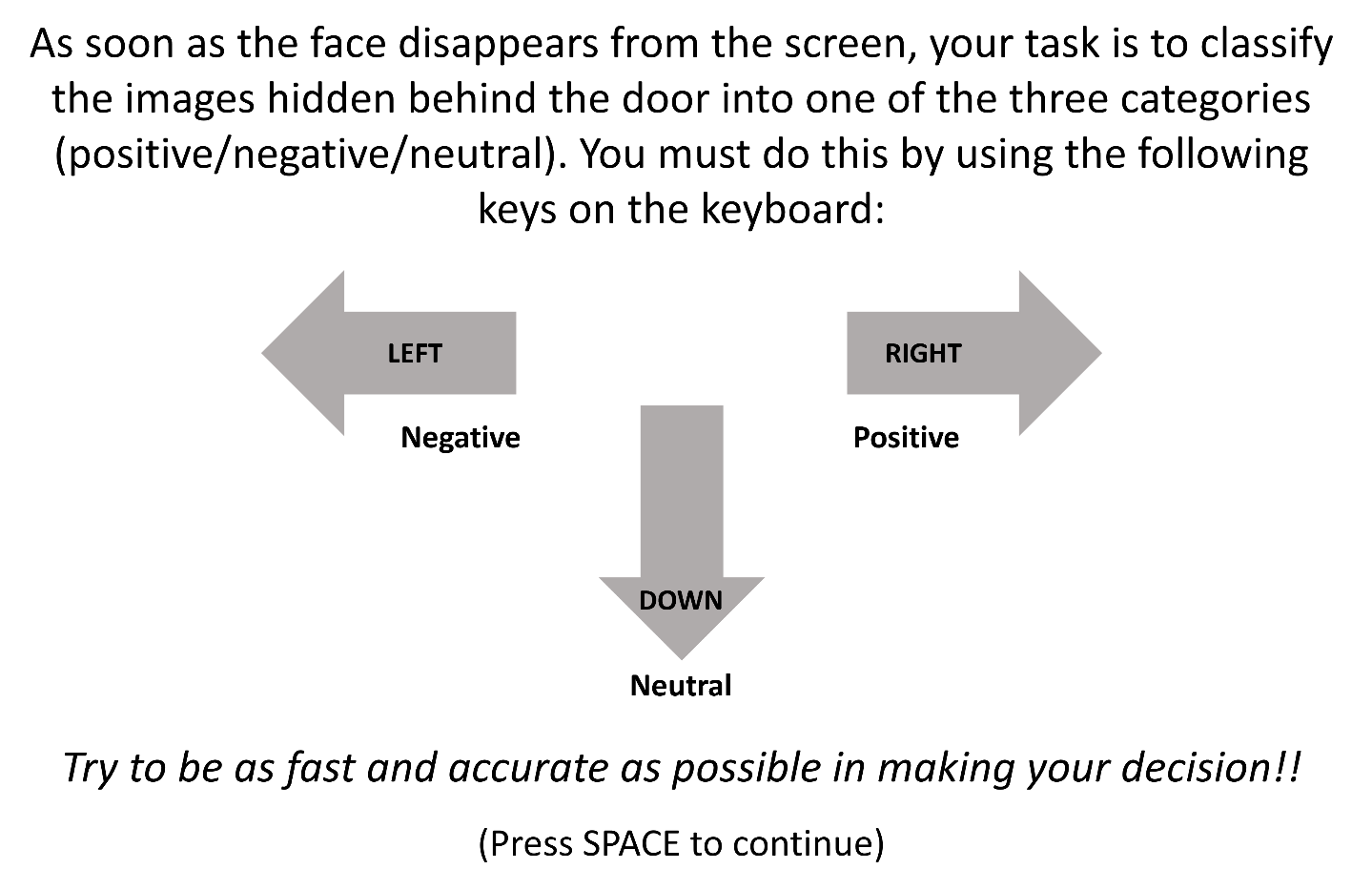
**

*** The keypress assignments for Negative and Positive will be counterbalanced and will be changed accordingly in the instructions for every participant**

**
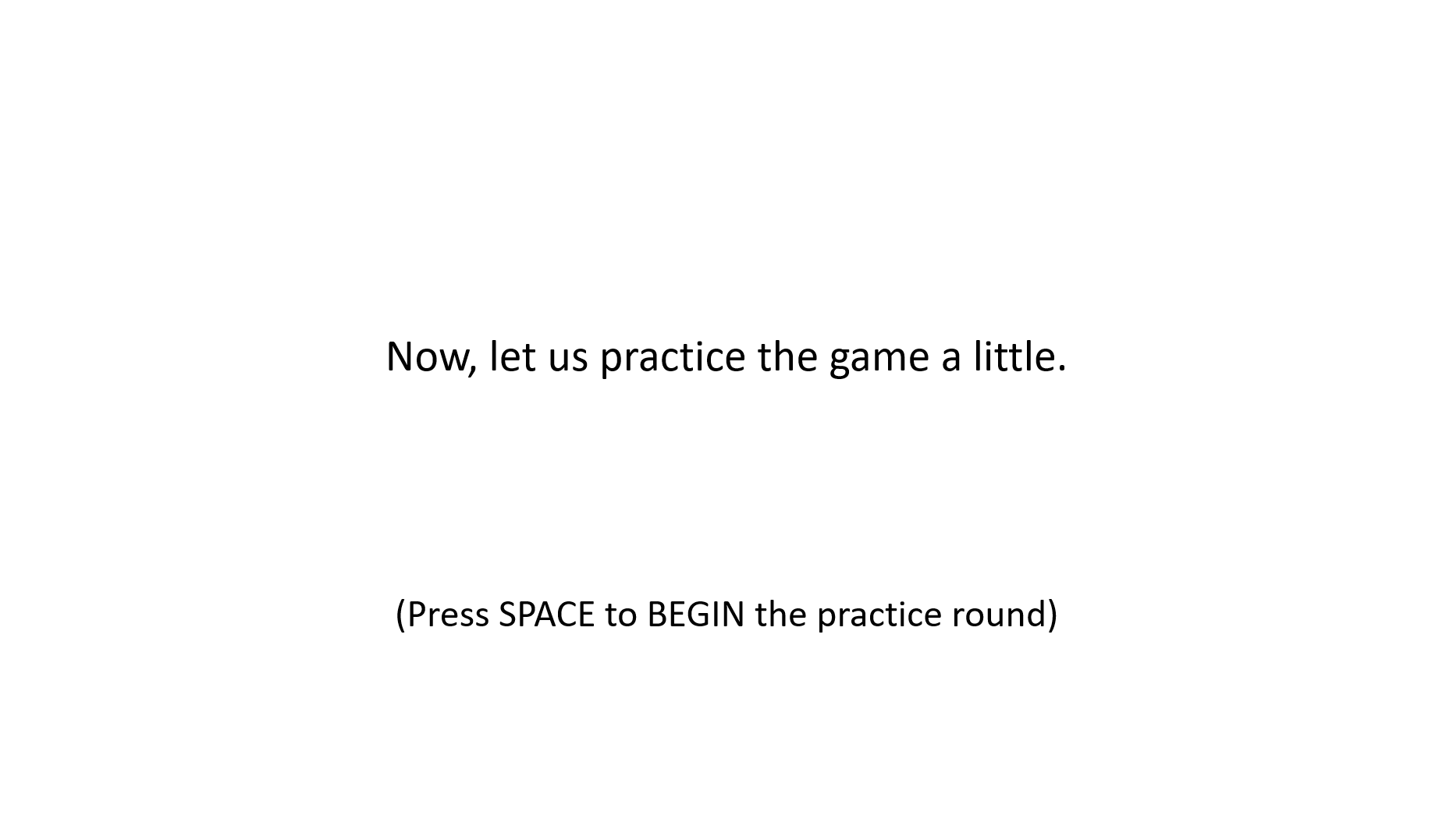
**

**
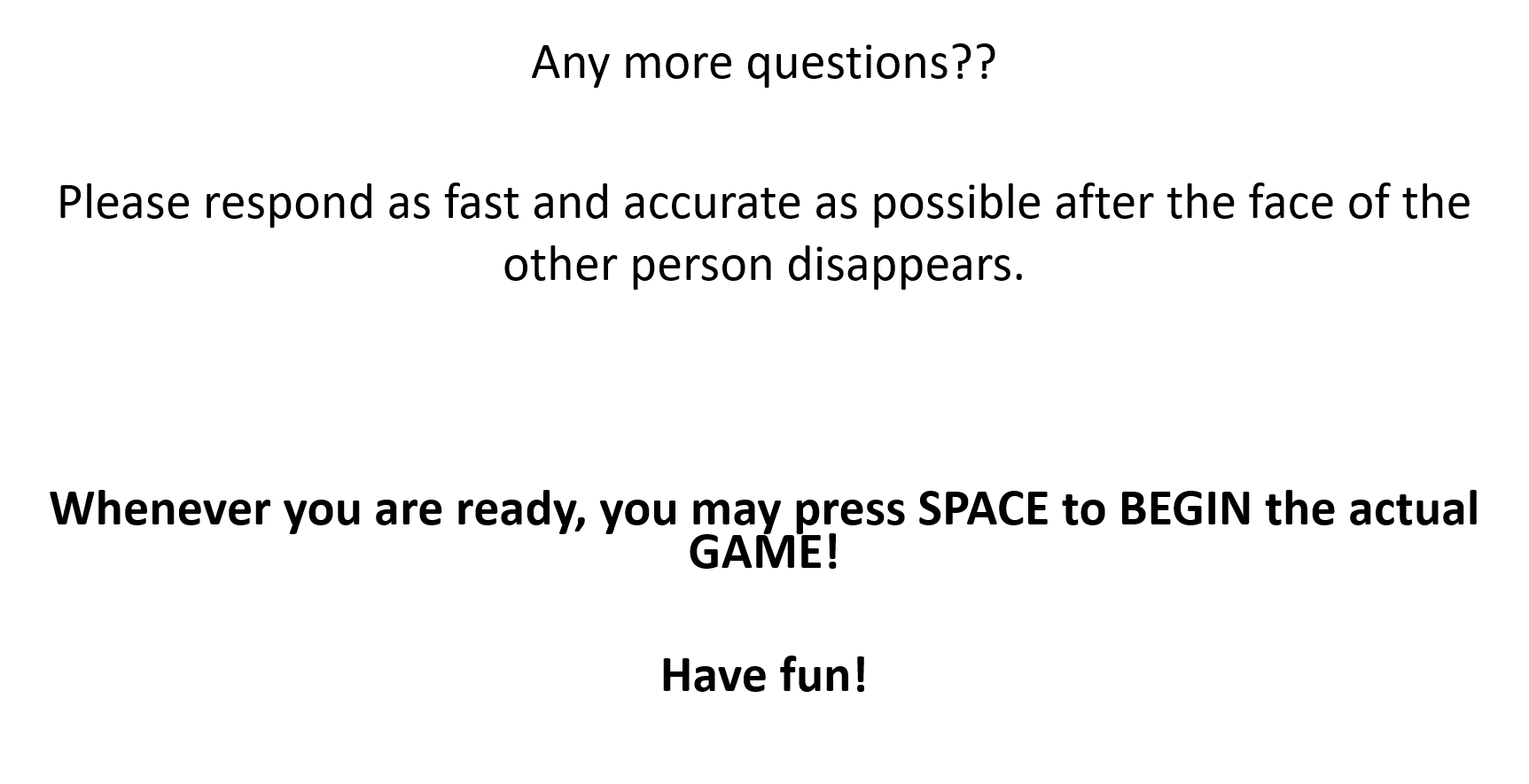
_____________________________________________________________________________________**

**Memory test immediately following the learning session (2 Blocks)**

**
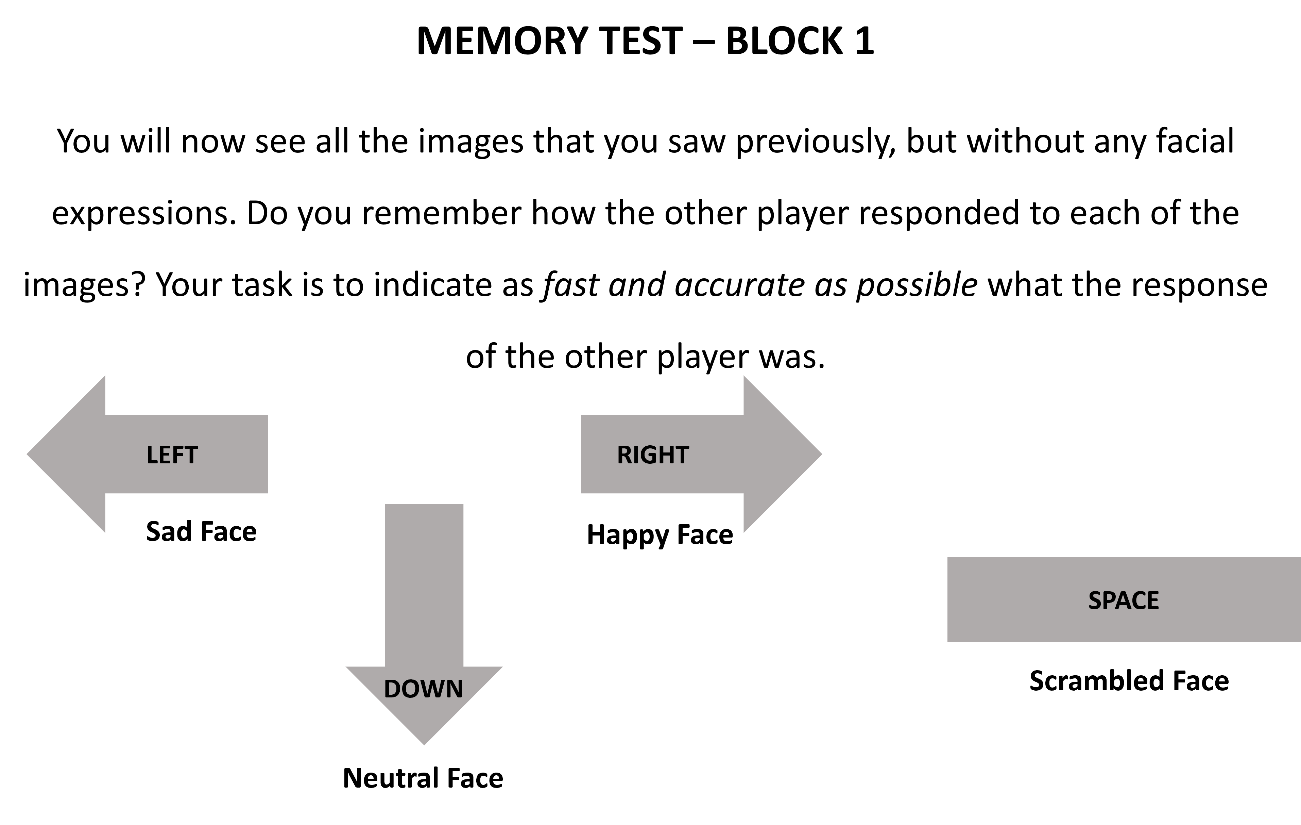
**

**
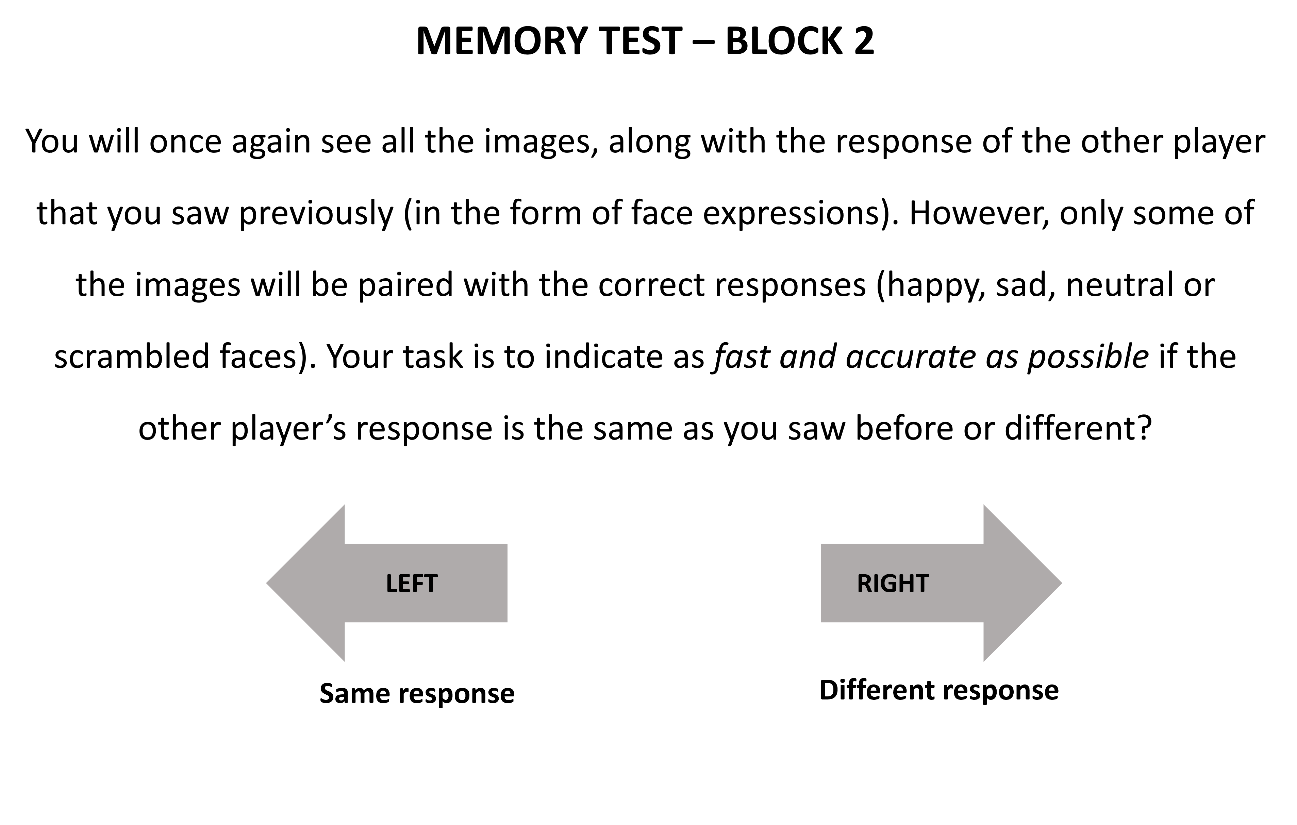
**

**Day2-Test Session: Old/New Classification Task**

**
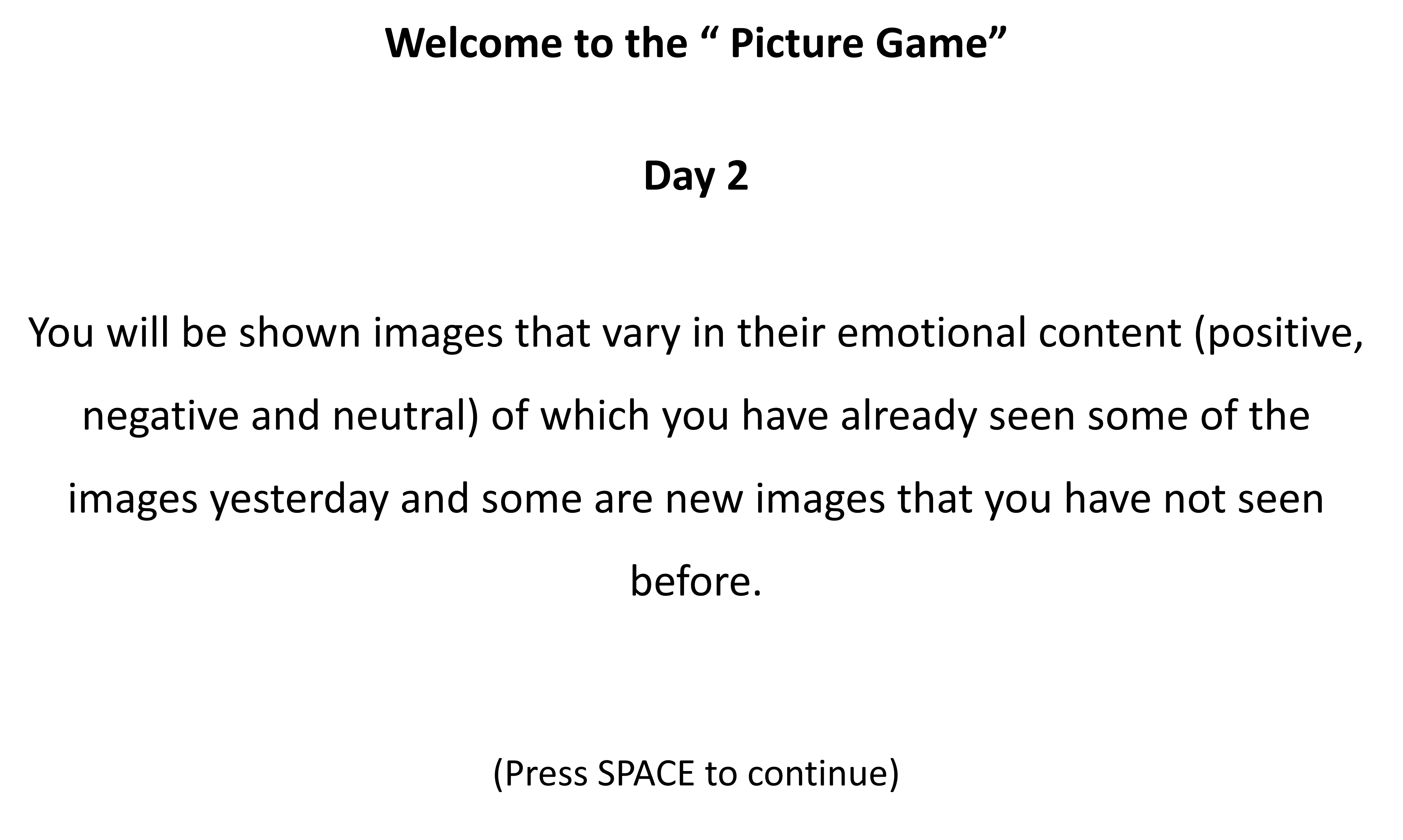
**

**
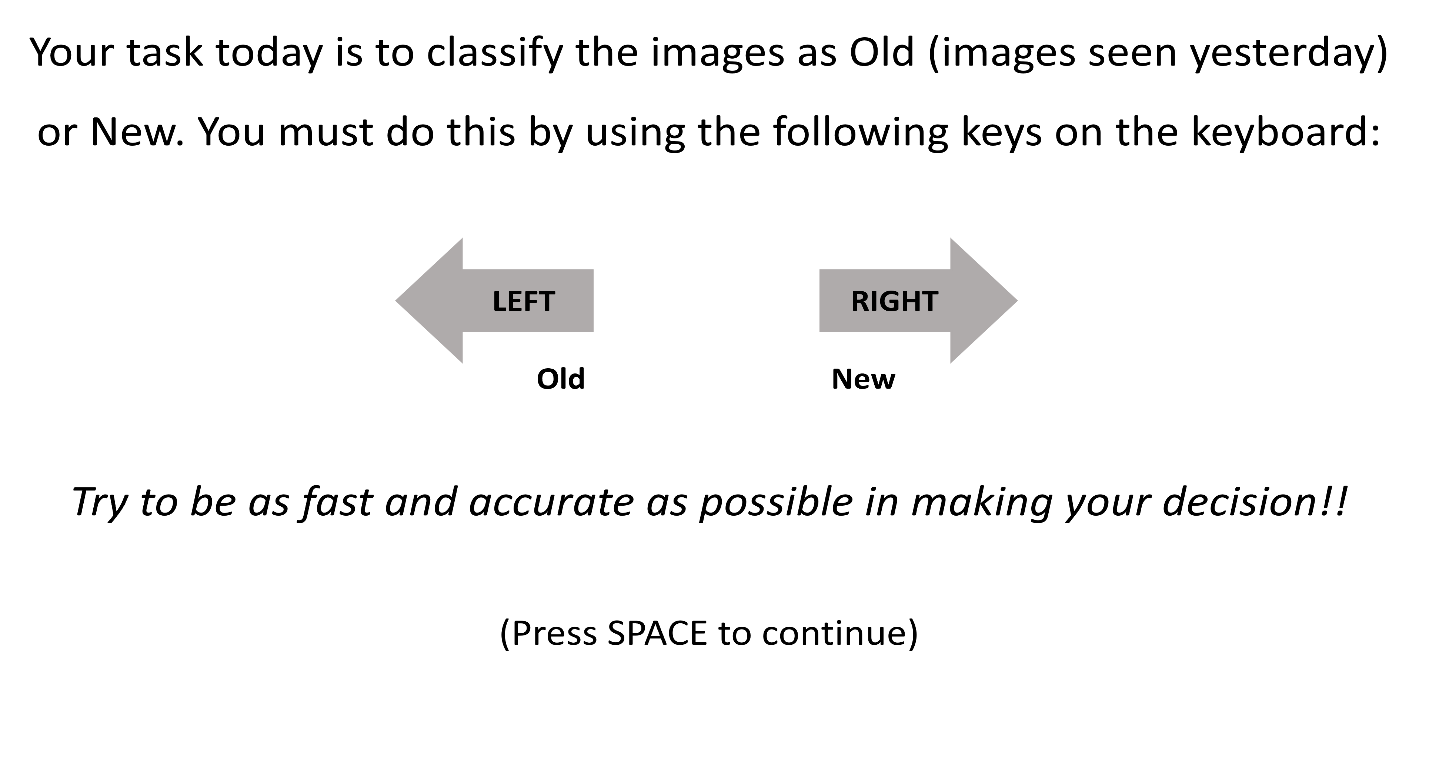
**

*** The keypress assignments for Negative and Positive will be counterbalanced and will be changed accordingly in the instructions for every participant**


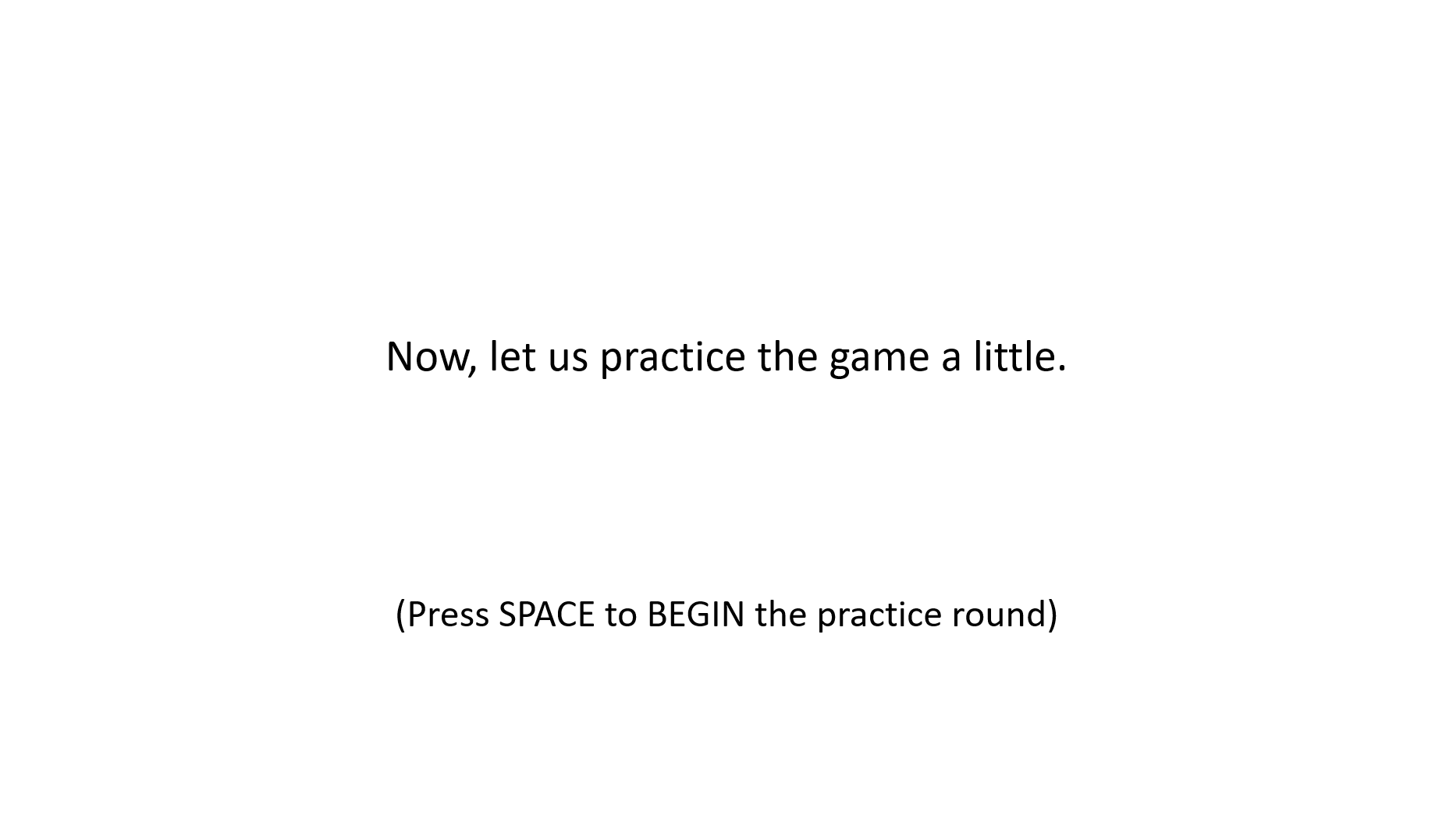


_______________________________________________________________________


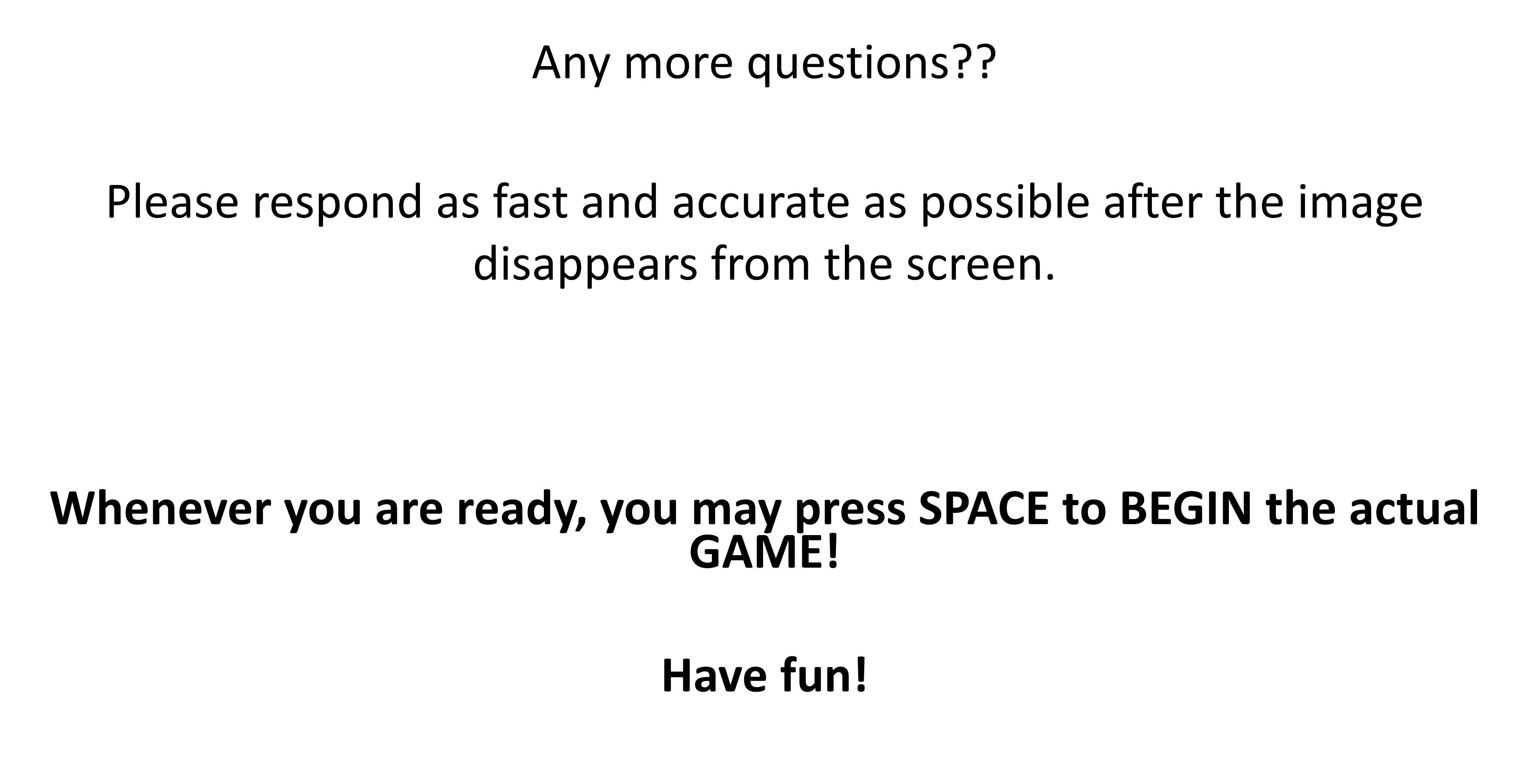

Supplement: Supplementary file 1 — Supplementary Material 1 [file 41598_2026_42906_MOESM1_ESM.docx]
